# In Vivo Tracking of Extracellular Vesicles by Nuclear Imaging: Advances in Radiolabeling Strategies

**DOI:** 10.3390/ijms21249443

**Published:** 2020-12-11

**Authors:** Sara Almeida, Liliana Santos, Amílcar Falcão, Célia Gomes, Antero Abrunhosa

**Affiliations:** 1 CIBIT/ICNAS—Institute for Nuclear Sciences Applied to Health, University of Coimbra, 3000-548 Coimbra, Portugal; sara.almeida@uc.pt (S.A.); liliana.santos.ca26@gmail.com (L.S.); amilcar.falcao@uc.pt (A.F.); 2 iCBR—Coimbra Institute for Clinical and Biomedical Research, Faculty of Medicine, University of Coimbra, 3000-548 Coimbra, Portugal; 3 CIBB—Center for Innovative Biomedicine and Biotechnology, University of Coimbra, 3000-548 Coimbra, Portugal; 4 CACC—Clinical Academic Center of Coimbra, 3000-075 Coimbra, Portugal

**Keywords:** extracellular vesicles, in vivo tracking, nuclear imaging, radiolabeling

## Abstract

Extracellular vesicles (EVs) are naturally secreted vesicles that have attracted a large amount of interest in nanomedicine in recent years due to their innate biocompatibility, high stability, low immunogenicity, and important role in cell-to-cell communication during pathological processes. Their versatile nature holds great potential to improve the treatment of several diseases through their use as imaging biomarkers, therapeutic agents, and drug-delivery vehicles. However, the clinical translation of EV-based approaches requires a better understanding of their in vivo behavior. Several imaging technologies have been used for the non-invasive in vivo tracking of EVs, with a particular emphasis on nuclear imaging due to its high sensitivity, unlimited penetration depth and accurate quantification. In this article, we will review the biological function and inherent characteristics of EVs and provide an overview of molecular imaging modalities used for their in vivo monitoring, with a special focus on nuclear imaging. The advantages of radionuclide-based imaging modalities make them a promising tool to validate the use of EVs in the clinical setting, as they have the potential to characterize in vivo the pharmacokinetics and biological behavior of the vesicles. Furthermore, we will discuss the current methods available for radiolabeling EVs, such as covalent binding, encapsulation or intraluminal labeling and membrane radiolabeling, reporting the advantages and drawbacks of each radiolabeling approach.

## 1. Introduction

Extracellular vesicles (EVs) are naturally heterogeneous nano-sized membrane-enclosed vesicles secreted by nearly all cell types in the body under both physiological and pathological conditions and can be detected in various body fluids such as blood, urine, milk, saliva, and central nervous system fluids [1].

Based on their biogenesis, size and membrane composition, EVs can be broadly categorized into three main groups: exosomes, microvesicles, and apoptotic bodies [2]. Exosomes are small vesicles of endocytic origin that are 50-150 nm in diameter and are formed from the inward budding of the limiting endosomal membrane of multivesicular bodies (MVBs) containing intraluminal vesicles (ILVs), which are released into the extracellular space upon MVBs fusion with the plasma membrane [3,4]. Microvesicles are heterogeneous with a size ranging from 100 to 1000 nm and are formed through the outward budding of the plasma membrane. They are released into the surrounding extracellular milieu by outward budding and fission with the cell membrane [5,6]. Apoptotic bodies are generally large, with a diameter of 1000–5000 nm and are released during the apoptotic phase of cell death, and they contain remnants of apoptotic cells (nuclear fractions and cytoplasmic organelles) that can be delivered to healthy recipient cells [7]. 

EVs are important mediators of cell-to-cell communication through the transfer and delivery of functionally active biological molecules, including nucleic acids in the form of DNA and RNAs, proteins, and lipids into the surrounding tissues or to distant recipient cells, where they can elicit a biologic response [8,9,10]. Intercellular communication through EVs seems to be involved in the regulation of multiple physiological processes and also in the pathogenesis of several diseases, including cancer, inflammation, and cardiovascular, neurological, and autoimmune pathologies [5]. Indeed, EVs load unique cargoes reflecting the origin and status of the donor cell, and they could potentially be used as imaging biomarkers for diagnostic and prognostic applications [11,12,13,14]. Beyond this, EVs travel safely in extracellular fluids and can cross biological barriers and penetrate dense structural tissues delivering endogenous cargo to target cells with high specificity and efficiency [8]. For these reasons, EVs became a topic of intense research also as potential vehicles for drug delivery in anti-tumor therapy, immunomodulation, and regenerative medicine [15]. Compared to organic or non-organic nanoparticles, EVs possess advantageous features, offering innate biocompatibility, low immunogenicity, high physicochemical stability, long-distance communication, and an intrinsic targeting ability to interact with cells via signal transduction and membrane fusion [13,16,17].

Despite the efforts and advances in this field, a major challenge hindering the use of EVs in a diagnostic or therapeutic context is the limited knowledge regarding their in vivo biological behavior in real time [18]. Determination of the biodistribution profile and circulation kinetics, along with the targeting ability to specific cells/tissues, route of uptake, and cargo delivery efficiency to recipient cells, is a prerequisite for an effective use of EVs in a therapeutic or drug-delivery context in biomedical applications [18,19,20].

Non-invasive molecular imaging tools can provide an accurate understanding of the in vivo behavior of exogenous EVs [21,22,23]. Among the different modalities available in preclinical research, nuclear imaging stands out from the others, mostly due to its high sensitivity and safety profile, and its enormous potential for clinical translation. 

Herein, we will provide an overview of the molecular imaging modalities available for the in vivo tracking of EVs, with a particular focus on nuclear imaging modalities, namely single-photon emission tomography (SPECT) and positron emission tomography (PET), and the current knowledge regarding the radiolabeling approaches for EVs.

## 2. Imaging Modalities for In Vivo Tracking of EVs 

Nowadays, several molecular imaging modalities are available for the non-invasive tracking of exogenous administered EVs in living organisms [24]. These approaches provide valuable insights on the functionality, viability, and trafficking of these vesicles in real time in a non-perturbed in vivo environment that are essential for an in-depth understanding of the dynamic interaction of EVs with their targets at both cellular and molecular levels. Such systems allow the exploitation of biological functions and mechanisms of EVs and potential applications in basic and translational life sciences in a broad spectrum of diseases [21,25]. 

Technically, tracking EVs in living organisms is quite challenging due to their small size, similar composition to body cells, and rapid dispersion in body fluids [26], and it requires highly sensitive and reliable techniques and EVs manipulation prior to their subsequent visualization. Moreover, it is fundamental to assure the membrane integrity and biological activity of EVs after labeling with exogenous probes [27].

The most common imaging technologies for in vivo tracking of EVs include optical-based imaging using fluorescence (FLI) and bioluminescence (BLI), magnetic resonance imaging (MRI), computed tomography (CT), and nuclear imaging, including SPECT and PET, each with their own advantages and limitations [21,25,28]. A summary of the main characteristics is listed in Table 1. 

Fluorescence and bioluminescence optical imaging rely on the detection of light within the visible light spectrum and have the advantage of high-throughput efficiency at low cost [26]. BLI requires plasmid transfection or the stable transduction of donor cells with reporter luciferases for the generation of bioluminescent signals, while FLI uses fluorescent protein or organic dyes to emit signals after excitation with an external source [21,31,32]. Exogenous near-infrared fluorescent (NIRF) dyes such as cyanine 7 (Cy7) and 1,1′-dioctadecyl-3,3,3′,3′-tetramethylindotricarbocyanine iodide (DIR) have been widely used as fluorescent imaging probes in living subjects due to their favorable optical properties (high signal-to-noise ratio, low autofluorescence of biological tissues, and deep tissue penetration) [25,29]. BLI has the advantage of high sensitivity and low background noise, but the inherently poor penetration depth and low spatial resolution makes it unsuitable for clinical translation [33]. The main advantages are the safe and straightforward use of non-ionizing light sources and their moderate cost-effectiveness.

Superparamagnetic iron oxide nanoparticles (SPION) and ultrasmall superparamagnetic iron oxide nanoparticles (USPION) are attractive probes for EV labeling and in vivo tracking by MRI because of their small size and biocompatibility [34,35]. Despite the exquisite spatial and temporal resolution of MRI, the low sensitivity of detection and the signal dependence on the surrounding tissues (magnetic relaxation of surround water protons and other nuclei in tissues) is a limiting factor for detecting the picomolar concentration of EVs that typically accumulated in the tissues [34,36]. CT has also been used to image EVs, but similarly to MRI, it has low sensitivity and requires the labeling of EVs with inorganic molecules that can accumulate in tissues [37].

Nuclear imaging, encompassing SPECT and PET, is a highly sensitive technique that uses gamma- or positron-emitting radionuclides as imaging probes and is at the forefront of the molecular imaging modalities [38]. The main advantages over the other imaging modalities are the high sensitivity, unlimited penetration depth, and accuracy of quantification, allowing the detection of probes typically in the pico- to nanomolar ranges [38,39], which make them suitable for the non-invasive tracking of EVs in living animals. These systems, although initially developed for clinical use, have been scaled down to provide the high-resolution imaging of small animals, which strengthens the translational potential of preclinical research in animal models [40].

SPECT imaging is based on the detection of single-photons emitted by gamma-emitting radionuclides with energies in the range of 30 to 300 keV and half-lives varying from hours to days such as technetium-99 m (^99m^Tc, t_1/2_ = 6h), indium-111 (^111^In, t_1/2_ = 2.8 days), or iodine-123 (^123^I, t_1/2_ = 13.2 h) [41].

PET is based on the detection of two time-coincident high-energy photons resulting from the decay of a positron-emitting radioisotope. The emitted positrons annihilate with nearby electrons in the tissue and produce a pair of 511 keV photons moving in the opposite directions providing higher resolution images with a 2- to 3-fold superior sensitivity over SPECT [39,42]. Some of the most widely used isotopes include fluorine-18 (^18^F, t_1/2_ = 110 min), gallium-68 (^68^Ga, t_1/2_ = 68 min), copper-64 (^64^Cu, t_1/2_ = 12.7 h), or zirconium-89 (^89^Zr, t_1/2_= 3.3 days) [36,38]. Both systems are commonly integrated with CT or MRI in hybrid devices to provide both molecular and anatomical details for an accurate localization of the imaging probes [21]. Apart from that, as most radionuclides are already in the clinic, there are fewer ethical and legal obstacles for clinical translation [21], which can accelerate the widespread use of EVs for clinical diagnosis and therapeutic applications.

## 3. Radiolabeling Methods for Extracellular Vesicles

**Figure 1:**
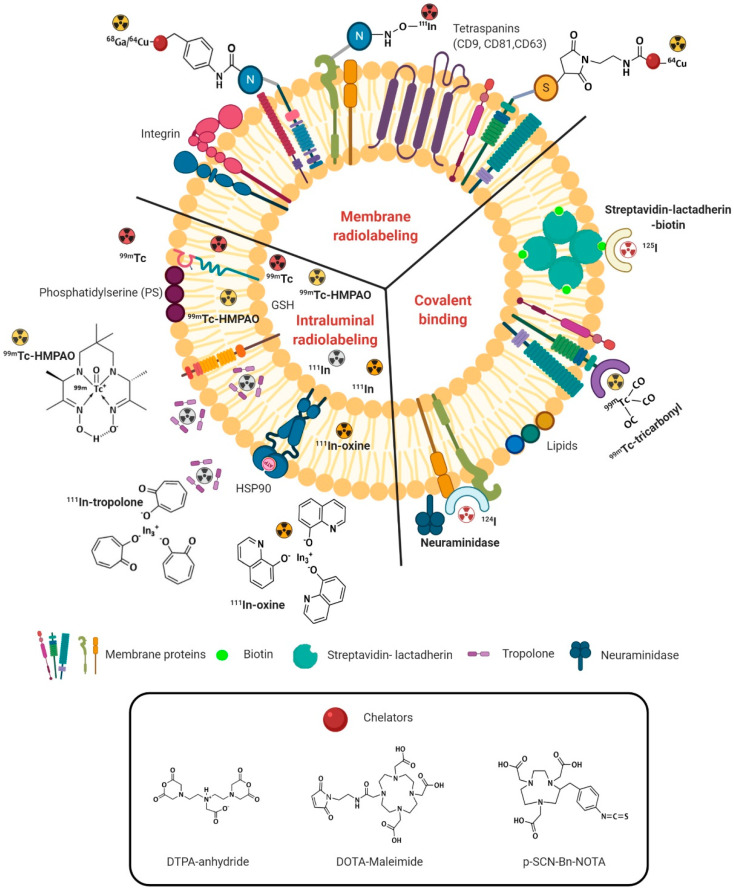
Schematic representation of the three radiolabeling methods of extracellular vesicles (EVs). Membrane radiolabeling can be carried out using bifunctional chelators (e.g., diethylene triamine pentaacetic acid (DTPA)-anhydride, 1,4,7,10-tetraazacyclododecane-1,4,7,10-tetracetic acid (DOTA)-maleimide, and p-SCN-Bn-NOTA (1,4,7-triazacyclononane-1,4,7-triacetic acid)) containing a functional group for covalent attachment to reactive amine (-NH2) or thiol (-SH) groups onto the surface of EVs and a metal-binding moiety for radiometal sequestration such as ^64^Cu, ^68^Ga, or ^111^In. Radionuclides can be encapsulated by intraluminal labeling using lipophilic ^111^In-oxine or ^99m^Tc-HMPAO complexes that cross spontaneously the EVs’ membrane and get into the vesicle lumen. Tropolone, a small hydrophobic molecule, is an alternative approach for ^111^In^3+^ shuttling into the exosomal lumen. Covalent binding is based on surface covalent bond formation between naturally reactive functional groups or non-native binding groups previously introduced in EVs with imaging probes. Streptavidin–biotin–lactadherin fusion protein, tyrosine residues in neuraminidase-treated EVs, and native amino acids present on the surface of EVs form stable complexes with radionuclides, such as ^124^I and ^99m^Tc.

Several strategies have been employed to label EVs with gamma- or positron-emitting radionuclides for SPECT and PET applications. The different approaches can be broadly classified into three categories: covalent binding, encapsulation or intraluminal labeling, and membrane radiolabeling, as illustrated in Figure 1. The principles of each technique will be addressed in the following sections, highlighting the limitations and advantages of each one that should be taken into consideration when choosing the radiolabeling method.

### 3.1. Covalent Binding 

At their surface, unmodified EVs contain reactive amine/carboxylic terminated phospholipids or transmembrane proteins available for covalent binding with imaging probes [43]. This labeling strategy is based on the formation of stable covalent bonds between those naturally reactive functional groups and probes conjugated to chemical groups that react with specific moieties onto the surface of EVs [30]. Many of these common bioconjugation reactions proceed under very mild conditions, thus avoiding membrane disruption or the denaturation of surface proteins, and due to the strength and stability of these chemical bonds, covalent labeled EVs are less prone to dissociation, which is a key factor in vivo applications [13]. 

Another strategy is to target non-native binding groups previously introduced in EVs through genetic engineering of donor cells. This approach was employed by Morishita et al. [44] in the radiolabeling of melanoma B16BL6 cells-derived exosomes with iodine-125 (^125^I) using the streptavidin–biotin system [44]. The B16BL6 donor cells were transfected with a plasmid vector encoding a fusion protein composed of Streptavidin (SAV, a protein that binds biotin with high affinity) and Lactadherin (LA, a protein known to locate to the outer surface of exosomes) to generate exosomes expressing SAV-LA. The engineered exosomes were further incubated with ^125^I-radiolabeled biotin derivative (3-^125^I-iodobenzoyl) norbiotinamide (^125^I-IBB) to obtain ^125^I-labeled B16BL6 exosomes. The SAV-LA modification did not induce significant alterations in the physicochemical properties or morphology of exosomes and resulted in radiochemically stable 1^25^I-labeled exosomes suitable for tissue biodistribution analysis in mice [44]. The genetic engineering of donor cells to endow exosomes with surface functional molecules such as biotin derivatives represents an efficient strategy for conjugation with radionuclides without interfering with native active sites; however, this is not applicable to all types of EVs.

Varga and collaborators [45] developed an outer-membrane labeling method using an organometallic technetium-99m (^99m^Tc(I))-tricarbonyl complex [^99m^Tc(CO)_3_(-H_2_O)_3_]^+^ for the radiolabeling of erythrocyte-derived EVs. The ^99m^Tc-tricarbonyl complex binds with a high affinity to several amino acids such as histidine, methionine, and cysteine of the surface proteins on EVs membranes by simple mixing with purified EVs at room temperature. This direct surface labeling of EVs represents a rapid and efficient way of producing ^99m^Tc-labeled EVs with an acceptable radiochemical yield and high in vivo stability. SPECT/CT imaging acquired after the intravenous administration of ^99m^Tc-labeled EVs in a mouse model showed high accumulation in the liver and spleen similar to previous fluorescence biodistribution imaging studies but distinct from that observed with free ^99m^Tc-tricarbonyl, demonstrating the reliability of the radiolabeling procedure and its potential application for the in vivo tracking of EVs by SPECT imaging [45].

Another direct radiolabeling method was recently reported by Royo and colleagues [46] using iodine-124 (^124^I), which is a long-lived positron emitting-radionuclide with a half-life of 4.2 days that enables the in vivo tracking of labeled EVs from hours to days after administration. This study was performed in neuraminidase-treated EVs derived from a hepatic mouse cell line (MLP29) to confirm the importance of the surface glycoproteins in the fate of these vesicles. The direct radioiodination with [^124^I]NaI was carried out through an electrophilic aromatic substitution on the tyrosine residues of proteins embedded on the surface of EVs, using pre-coated iodination tubes. The radiochemical yield after purification was about 20% with a loss in EVs protein content of 5%. The stability in a physiological saline solution remained quite stable with a release of the free radionuclide inferior to 10% over a 72 h period, allowing accurate and quantitative tracking of the radiolabeled EVs by PET over a longer time in living mice [46]. A similar approach was performed by Rashid et al. [47], using ^131^I and iodination beads to assess in vivo the biodistribution of exosomes from diverse cellular origins, by SPECT/CT in living mice. They also confirmed the high stability of the ^131^I-radiolabeled exosomes after serum challenging at 24 h [47]. More recently, González et al. [48] developed a radiochemical method using milk-derived exosomes based on reduced ^99mc^Tc (IV) and commercial pertechnetate ^99mc^Tc (VII) for in vivo SPECT/CT imaging. Redox conditions were optimized to reduce [^99mc^Tc]NaTcO_4_ in order to obtain a more stable oxidation state +4, allowing the reaction with the exosomes. This ^99m^Tc labeling method is based on its coordination with the phosphonate groups present in the membrane’s exosomes, which enable a passive surface labeling. The stability of this passive incorporation remained stable in phosphate-buffered saline (PBS). On the other side, the incubation of exosomes with commercial ^99mc^Tc (VII) did not occur, showing that the oxidation state is crucial to radiochemical reaction [48].

### 3.2. Encapsulation or Intraluminal Radiolabeling

Radionuclides can alternatively be encapsulated and trapped into the lumen of EVs. This strategy also referred to as encapsulation or intraluminal radiolabeling was developed to avoid modifications on the surface of EVs. This can be performed by passive diffusion using hydrophobic probes that spontaneously cross the membrane and get into the vesicle lumen or through active loading strategies that require the use of techniques, such as electroporation or sonication that transiently disrupt the lipid bilayer to facilitate the loading of cargo into EVs [11,13]. Although more effective, these techniques can induce structural distortion of the membrane and carry the risk of aggregation or fusion of the vesicles, which might give rise to artifacts in the in vivo imaging [30]. Another strategy is the encapsulation via a specific transporter on the exosomal membranes, such as the GLUT1 glucose transporter for radiolabeled glucose analogs, such as ^18^F-fluoro-2-deoxy-D-glucose (FDG), but differences in the distribution and variable expression of receptors might lead to the uneven loading of imaging probes.

Hwang et al. [49] developed a radiolabeling method for macrophage-derived exosome-mimetic nanovesicles (ENVs), which share biological features with naturally derived exosomes, using the ^99m^Tc radioisotope attached to hexamethylpropyleneamineoxime (HMPAO) for SPECT/CT imaging. ^99m^Tc-HMPAO is an uncharged low molecular weight and a highly lipophilic radiotracer that easily diffuses across the cell membranes, being widely used for cell labeling. HMPAO is first mixed with ^99m^Tc for the formation of the lipophilic ^99m^Tc-HMPAO complex and then incubated with ENVs under mild condition for not changing their properties. After diffusion across the lipid bilayer, ^99m^Tc-HMPAO reacts with the sulfhydryl groups of glutathione (GSH) within the lumen and is converted into its hydrophilic form, remaining trapped irreversibly inside the ENVs. The final product was obtained with a radiochemical purity of nearly 100%, and the stability studies in serum showed that approximately 90% of entrapped ^99m^Tc in ENVs persisted within the vesicles until 5 hr, allowing biodistribution studies over long periods. Importantly, the average size distribution using nanoparticle tracking analysis (NTA) did not change significantly after labeling or the expression levels of CD63, which is an exosome-specific protein. In fact, SPECT/CT imaging studies in mice of ^99m^Tc-HMPAO-labeled ENVs showed a biodistribution pattern in mice similar to natural EVs, being taken mainly by the liver and spleen [49]. A limiting factor for this radiolabeling method could be the low amount of GSH in EVs when compared with cell lysates [50].

A similar method was described by Smyth et al. [51], using ^111^Indium (^111^In), a gamma-emitting radioisotope with 2.8 days half-life and 8-hydroxiquinoline (oxine) as a carrier. The radiolabeling mechanism is thought to involve an exchange reaction between the carrier and exosome’s components. ^111^In forms an uncharged pseudo-octahedral N_3_O_3_ complex with oxine that diffuses freely through the lipid bilayer of exosomes, and once inside, ^111^In^3+^ ions detach from the oxyquinoline and form a stable complex with the vesicle’s components. As for HMPAO, the radiolabeling conditions occur under mild conditions not modifying the size or function of exosomes. A disadvantage could be the radiation exposure to critical organs and the whole body to ^111^In-oxine, which is substantially higher than that from ^99m^Tc-HMPAO due to the higher energy *γ*-radiation (171 and 245 keV) and the longer half-life (2.8 days) of ^111^In [51]. 

More recently, Faruqu et al. [52] proposed an alternative approach to shuttle ^111^In^3+^ ions inside the exosomal lumen by using tropolene instead of oxine. Tropolone is a small hydrophobic molecule that also forms a complex with ^111^In (as ^111^InCl_3_) and diffuses across the exosomal membrane into the lumen. As the interaction between ^111^In^3+^ and tropolone is not particularly strong, ^111^In^3+^ detaches from the [^111^In]-tropolone and exchanges with proteins and nucleic acids within the exosomal lumen. Free tropolone molecules leave exosomes and the ^111^In^3+^ remains entrapped within the lumen, thereby resulting in radiolabeled exosomes. The lack of biomolecules for exchange in exosomes can contribute to the low radiolabeling efficiency and stability. In this case, the unchanged ^111^In^3+^ in the form of [^111^In]-tropolene leaves the exosomal lumen and forms an equilibrium in terms of its concentration within and the outside of the lumen that might lead to a misleading interpretation of the in vivo biodistribution data. In fact, the stability of intraluminal radiolabeled exosomes was found to be 43% in PBS and 14.2% in 50% serum at 24 h post-incubation [52], leading the authors to co-opt for a membrane radiolabeling approach (discussed in the next section) for whole-body SPECT-CT imaging studies.

Gangadaran et al. [53] employed another radiolabeling strategy with ^99m^Tc for large-scale production exosome mimetics (EMs) derived from red blood cells (RBCs). Engineered EMs were incubated with 0.01% Tin (II) chloride, which is an effective reducing agent, followed by ^99m^TcO_4_^−^. Tin chloride reduces Tc (VII) to a lower oxidation state presumably +3 to allow the reaction with the hemoglobin inside the EMs. This ^99m^Tc labeling procedure resulted in excellent radiochemical purity (almost 100%), without the need of column purification methods, which may cause the loss of some exosomes in the process, and it does not affect the size or shape of RBCs-EMs. Moreover, RBC-EMs retained 97% of the entrapped ^99m^Tc for up to 3 h and even after 24 h, the radiochemical purity was 93%. The in vivo gamma camera images allowed the visualization of ^99m^Tc-RBCs-EMs in deep organs such as the liver and spleen, which is distinct from the biodistribution of free ^99m^TcO_4_^-^ [53]. 

### 3.3. Membrane Radiolabeling 

Another strategy is based on the surface functionalization of EVs with bifunctional chelators (BFCs). Bifunctional chelators are dual molecules containing a functional group for covalent attachment to a reactive amine, thiol, or carboxylic group onto the surface of EVs and a metal-binding moiety for radionuclide sequestration [43]. This click chemistry-based method has proved effective while affording high yielding under simple mild reaction conditions when using the proper matching chelator to the radiometal [54,55,56]. There are several BFCs commercially available (e.g., diethylene triamine pentaacetic acid (DTPA); 1,4,7,10-tetraazacyclododecane-1,4,7,10-tetracetic acid (DOTA); 1,4,7-triazacyclononane-1,4,7-triacetic acid (NOTA)) that have been used for conjugation with radiometals commonly used in basic and clinical research for imaging purposes.

Faruqu et al. [52] demonstrated the feasibility of this approach in melanoma-derived exosomes using a bifunctional chelator DTPA-anhydride that forms a stable covalent bond in an amine-dependent reaction. Free amine groups act as nucleophiles that attack anhydrides on DTPA, resulting in exosomes with DTPA covalently attached on their surface that rapidly form stable complexes with the radioisotope ^111^In^3^. The radiolabeling efficiency was superior to that obtained with the intraluminal labeling and SPECT/CT imaging analysis confirmed the in vivo stability without affecting their biological properties, including the expression of CD9 and CD63 exosomal markers [30]. 

Recently, our group reported a two-step surface modification method for the radiolabeling of small extracellular vesicles (SEVs) from human umbilical cord blood mononuclear cells (hUCB-MNCs) with ^64^CuCl_2_ for PET/MRI imaging [57]. The method includes the initial conjugation of the metal chelator DOTA on the surface of SEVs followed by the complexation with ^64^CuCl_2_. The free thiol groups on the surface of SEVs reacted under mild reaction conditions pH 7 with the maleimide group present in the DOTA chelator. These SEVs–DOTA conjugates react with ^64^Cu and form a kinetically inert and thermodynamically stable octahedron complex. The purity of radiolabeled SEVs, after removal of the non-complexed metal chelator DOTA and free copper, was 100%, and it remained stable in serum and PBS for at least 24 h. Importantly, no signs of aggregation or significant changes in terms of surface receptor proteins, morphology, size, or charge of SEVs were registered, confirming the feasibility of the surface labeling strategy. In vivo studies in mice confirmed the stability of the complex as indicated by the residual leaching of ^64^Cu (< 5%) from the chelator and the suitability for in vivo PET tracking and organ-specific biodistribution of SEVs [57].

Shi et al. [58] proposed another approach based on the use of the bifunctional chelator 2-S-(4-isothiocyanatobenzyl)-1,4,7-triazacyclononane-1,4,7-triacetic acid (p-SCN-Bn-NOTA) for ^64^Cu radiolabeling. The novelty of this method was the PEGylation on the surface of exosomes with the N-hydroxy succinimidyl (NHS) functionalized polyethylene glycol 5k (mPEG_5k_-NHS) through a nucleophilic attack to improve the pharmacokinetic profile and tumor accumulation. The ^64^Cu-labeled PEGylated exosomes rapidly complexed with ^64^CuCl_2_ under mild conditions, showing a high radiolabeling yield. This approach endowed exosomes with a superior in vivo pharmacokinetic profile resulting in enhanced tumor uptake as compared with native exosomes without a surface coating. Recently, Jung et al. [59] described a similar method for 4T1 breast cancer-derived exosome radiolabeling, using the same chelator p-SCN-Bn-NOTA of the radioisotopes ^64^Cu and ^68^Ga. Exosomes were also labeled with Cy7, which is a near infra-red fluorescence dye for optical imaging. A comparative analysis demonstrated that optical imaging only detected exosomes in one metastatic site (brachial lymph node), whereas PET clearly showed their localization in two different metastatic sites (axillary and brachial lymph nodes), proving the higher sensitivity of this imaging modality. Additionally, this strategy enables the quantitative information about the in vivo biodistribution of exosomes.

A summary of the radiolabeling methods described above are listed in Table 2.

## 4. Conclusions

The in vivo tracking of EVs is crucial for a better understanding of their role in physiological and pathological conditions. This knowledge is essential for the development of reliable EVs-based therapeutic and diagnostic tools. Nuclear imaging-based techniques such as PET and SPECT are highly attractive to serve this purpose owing to their quantitative nature, unlimited penetration depth, improved resolution and unparalleled sensitivity.

In this review, we summarized the various radiolabeling strategies used to incorporate radioisotopes onto EVs for biomedical applications. Several methods are discussed, including covalent binding, intraluminal labeling, and membrane radiolabeling. Key factors such as radiolabeling efficiency and stability in biological environments are discussed, as those are critical for an accurate characterization of the in vivo behavior of EVs within the timeframe of their biological half-life. 

The strategies described demonstrate how molecular imaging with PET or SPECT can be useful to guide the development of biomedical applications of EVs for medical diagnosis and treatment in the ever-evolving field of nanotechnology. 

## Figures and Tables

**Table 1:** Characteristics of non-invasive molecular imaging modalities [28,29,30].

Imaging Modality	Detection	Spatial Resolution	Depth Penetration	Temporal Resolution	Sensitivity (M)	Examples
**FLI and BLI**	visible light	2–5 µm	1–2 cm	s to min	10^−9^–10^−12^	GFP, Luciferase
**MRI**	radiofrequency	25–100 µm	no limit	min to h	10^−3^–10^−5^	SPION
**CT**	X-rays	50–200 µm	no limit	min	-	Gold nanoparticle
**PET**	pairs of γ-rays (511 keV)	2–4 mm	no limit	10 s to min	10^−11^–10^−12^	^64^Cu-DOTA
**SPECT**	single γ-ray (140 keV)	4–6 mm	no limit	min	10^−10^–10^−11^	^99m^Tc-HMPAO

BLI: bioluminescence imaging; CT: computed tomography; DOTA: 1,4,7,10-tetraazacyclododecane-1,4,7,10-tetracetic acid; FLI: fluorescence imaging; GFP: green fluorescent protein; HMPAO: hexamethylpropyleneamineoxime; MRI: magnetic resonance imaging; PET: positron emission tomography; SPECT: single-photon emission computed tomography; SPION: Superparamagnetic iron oxide nanoparticles.

**Table 2:** Tracking of extracellular vesicles biodistribution using nuclear imaging strategy.

Labeling Method	Nuclear Imaging	Radionuclide	EVs (Markers)	Origin	Mouse Strain	Dose	Admin. Route	In Vitro Stability	Ref.
Covalent binding	-	^125^I-IBB	Exosomes	B16BL6	BALB/c	37 KBq (4 µg)	I.V.	PBS 20% FBS	[44]
	SPECT/CT	^99m^Tc-tricarbonyl complex	EVs	RBCs	BALB/c	15 ± 2 MBq	I.V.	-	[45]
	PET	[^124^I]NaI	EVs	MLP29	BALB/cJRj	1.8 ± 0.5 MBq (120 ng) 0.6 ± 0.2 MBq (40 ng)	I.V. Hock	NaCl	[46]
	SPECT/CT	^131^I	Exosomes (CD9, CD63)	4T1; AT3	BALB/c, C57BL/J6	350 ± 50 µCi	I.V.	20% FBS	[47]
	SPECT/CT	^99m^Tc	Exosomes	Goat milk	BALB/c	140–170 µCi (12 µg) 190–340 µCi (19 µg) 310–350 µCi (18 µg)	I.N. I.P. I.V.	PBS	[48]
Encapsulation	SPECT/CT	^99m^Tc-HMPAO	ENVs (CD63)	Raw 264.7	BALB/c	7.4–14.8 MBq (29–64 µg)	I.V.	Serum or PBS	[49]
	-	^111^In-oxine	Exosomes (HSP 70, 90, 27; CD9)	MCF-7 PC3	Nude Nu/J	7.2 µCi (32 µg) 7.6 µCi (30 µg)	I.V.	-	[51]
	SPECT/CT	^111^In via tropolone	Exosomes (CD81, CD9)	B16F10	C57BL/6; NSG	5–10 MBq (1 × 10^11^ part.)	I.V.	50% FBS or PBS	[52]
	Gamma camera	^99m^Tc	Exosome mimetics	RBCs	C57BL/6	37 MBq	I.V.	PBS 20% FBS	[53]
Membrane radiolabeling	SPECT/CT	^111^In-DTPA	Exosomes (CD81, CD9)	B16F10	C57BL/6; NSG	5–10 MBq (1 × 10^11^ part.)	I.V.	50% FBS or PBS	[52]
	PET/MRI	^64^Cu-DOTA	SEVs (CD9, CD63, CD45)	hUCB-MNCs	C57BL/6J	100–150 µCi (2.5–3.5 × 10^10^ part.)	I.V.	PBS, serum	[57]
	PET	^64^Cu-NOTA-PEG	Exosomes	4T1	BALB/c	50 µCi	I.V.	PBS or 25% mouse serum	[58]
	PET	^64^Cu (or ^68^Ga)-NOTA	Exosomes	4T1	BALB/c nu/nu	0.74 MBq	I.V. S.C.	10% exo-free FBS	[59]

CT: computed tomography; DOTA: 1,4,7,10-tetraazacyclododecane-1,4,7,10-tetracetic acid; DTPA: diethylene triamine pentaacetic acid; ENVs: exosome-mimetic nanovesicles; exo-free: exosome-depleted; FBS: fetal bovine serum; HMPAO: hexamethylpropyleneamineoxime; HSP: heat shock proteins; hUCB-MNCs: human umbilical cord blood mononuclear cells; I.P.: intraperitoneal injection; I.V.: intravenous injection; MRI: magnetic resonance imaging; NaCl: sodium chloride; NOTA: 1,4,7-triazacyclononane-1,4,7-triacetic acid; NSG: NOD SCID gamma mice; PBS: phosphate-buffered saline; RBCs: red blood cells; S.C.: subcutaneous injection; SEVs: small extracellular vesicles; SPECT: single-photon emission computed tomography; ^125^I-IBB: (3-^125^I-iodobenzoyl) norbiotinamide.
